# Fallopian tube recanalization for the management of infertility

**DOI:** 10.1186/s42155-023-00356-z

**Published:** 2023-03-13

**Authors:** Anne Roberts

**Affiliations:** University of California, San Diego, 9300 Campus Point Drive, La Jolla, CA 92037 USA

**Keywords:** Fallopian tube occlusion, Selective salpingography, Infertility, Recanalization

## Abstract

Infertility is a world-wide problem, defined as failure to achieve pregnancy after 12 months of regular unprotected sexual intercourse. There are multiple causes for infertility involving both male and female factors. Fallopian tube occlusion is a common reason for female infertility. The initial attempts to treat proximal obstruction involved the use of a whalebone bougie positioned in the uterine cornua to dilate the proximal tube by Smith as early as 1849. Fluoroscopic fallopian tube recanalization for the treatment of infertility was first described in 1985. Since that time, there have been over 100 papers describing various methods for recanalization of occluded fallopian tubes. Fallopian tube recanalization is a minimally invasive procedure which is performed on an outpatient basis. It should be a first line therapy for patients with proximal occlusion of fallopian tubes.

## Background

Infertility is defined as a disease characterized by the failure to establish a clinical pregnancy after 12 months of regular, unprotected sexual intercourse in a woman under age 35, or after 6 months in a woman over the age of 35 (Carson and Kallen 2021; Marlow et al. 2021). Infertility is a common problem throughout the world. It is estimated that more than 186 million people suffer from infertility, the majority being residents of developing countries (Vander Borght and Wyns 2018). Infertility may be primary or secondary. Primary infertility is defined as a woman who has never had a clinical pregnancy (Vander Borght and Wyns 2018). Secondary infertility is the inability to establish a clinical pregnancy but the woman has previously been diagnosed with a. clinical pregnancy (Vander Borght and Wyns 2018). There are both male and female causes for infertility with the most common causes of female infertility being ovulatory dysfunction and tubal disease (Carson and Kallen 2021). Tubal infertility is estimated to account for between 11 and 67% of infertility diagnoses (Carson and Kallen 2021). Tubal infertility can be caused by internal blockage of the fallopian tubes, abnormalities of the internal tube from inflammation or infection, or pelvic adhesions which prevents normal functioning of the fallopian tubes. The blockage of the fallopian tubes may be proximal, in which case, the blockage is commonly composed of debris which forms a plug in the proximal tube. If the blockage is more distal, then the cause is usually previous infection, which may present as a hydrosalpinx with dilatation of the fallopian tube, and no spill from the tube on hysterosalpingogram (HSG). If the fallopian tube is blocked proximally, then selective recanalization of the tubes may be effective in opening the fallopian tube allowing for potential fertility.

## Fallopian tube occlusion

Fallopian tube disease is thought to account for 11–67% of all cases of infertility (Carson and Kallen 2021). On examining the causes of tubal infertility, 10–25% maybe due to proximal tubal occlusion with otherwise normal pelvic anatomy (Al-Omari et al. 2018; Sulak et al. 1987; Allahbadia and Merchant 2010). The proximal tube is described in the surgical literature as the uterotubal junction (UTJ). It is defined as the part of the tube that marks the transition area between the intramural (interstitial) and isthmic portions (Musich and Behrman 1983). The diameter of the tubal lumen in the proximal 4–5 cm is 1 mm or less (Musich and Behrman 1983; Thurmond et al. 2000). The cornual portion of the uterus and tube is very vascular because of network of vessels coursing through this area (Musich and Behrman 1983). The small caliber, thick muscular wall, and reduced proportion of ciliated cells in the epithelium of the proximal tube predispose this segment to blockage (Al-Omari et al. 2018; Allahbadia and Merchant 2010).

The cause of the proximal tubal obstruction maybe due to infection and subsequent inflammation, endometriosis, polyps, or salpingitis isthmica nodosum (SIN), all of these may lead to fibrosis of the oviduct (Sulak et al. 1987; Allahbadia and Merchant 2010; Kohi 2021). However, in some women the blockage may be caused by a “plug” of amorphous material in the tubal lumen. Histologically this represents a cast of partially organized inflammatory exudate, containing histiocytic-like cells of endometrial stroma or mesothelial origin, with mucous, and occasionally calcification (Sulak et al. 1987; Allahbadia and Merchant 2010). In a study of women undergoing hysterosalpingograms (HSG) it was found that 40% of women with proximal occlusion had mucous plugging or debris, and another 20% had uterotubal spasm (Marlow et al. 2021; Al-Omari et al. 2018). It is known that the pregnancy rate following HSG is 13–55% (Sulak et al. 1987) The increase in fertility following an HSG maybe due to the pressure created in the uterine cavity and then transmitted through the fallopian tubes dislodging the amorphous plug from the proximal tube. However, with some HSGs, despite pressurization of the uterine cavity to a point where the woman has significant discomfort, there is no filling of the proximal tube. In these cases, a selective transcervical catheterization and recanalization of the fallopian tube may establish patency.

The procedure of transvaginal fluoroscopically guided tubal recanalization was first described in 1985 by Platia and Krudy (Platia and Krudy 1985) They reasoned that even though considerable hydraulic pressure could be applied to the uterine cavity, that the net force transmitted to the interstitial oviduct would be quite weak because of its small cross-sectional area (Platia and Krudy 1985). They went on to describe a case of a 32 year old woman who had primary infertility of 1 ½ years duration. The patient had a HSG which showed partial patency of the right tube, and complete obstruction on the left. The patient underwent laparoscopy with injection of methylene blue dye demonstrating bilateral tubal obstruction with an otherwise normal appearing pelvis. The patient then had a second HSG which was the same as the first. A 3 French catheter was then inserted through the cervix and placed against the tubal ostium. An injection of contrast demonstrated the same area of obstruction. Then a 0.018 guidewire was placed through the catheter into the fallopian tube. The guidewire was removed, and a repeat injection demonstrated filling of the tube and spill into the peritoneum. The same maneuvers were performed on the left, but on the left side there was significant resistance to passage of the guidewire, and this tube was not recanalized. The patient ended up conceiving during her third cycle after the recanalization although the pregnancy ended with a 5 week spontaneous abortion (Platia and Krudy 1985).

In 1987, Thurmond, et al. described a series of 7 patients who had interstitial fallopian tube obstruction (IFTO), another term for proximal tubal obstruction, on HSG. Two of these patients had laparoscopy or laparotomy during which the feasibility and safety of transcervical tube probing with an 0.018 -in. guidewire was evaluated (Thurmond et al. 1987). Direct observation of the tube during guidewire advancement showed its smooth passage through the tube. The guidewire straightened the tube but did not perforate or damage the tube and an HSG follow-up showed patency of the recanalized tube (Thurmond et al. 1987). The other five patients had the procedure performed with fluoroscopic guidance. The procedure described is quite similar to the procedure described by Platia and Krudy in their paper (Platia and Krudy 1985). A Foley catheter which had an end hole placed through it allowed a 5 French catheter was placed into the uterus. The 5 French catheter was shaped with steam so there was a gentle 30-degree curve. The 5 French catheter was placed into the ostium of the fallopian tube and a selective salpingogram was performed. If no filling of the tube was demonstrated, then a 3 French catheter containing a 0.018″ guidewire was placed through the Foley catheter. The guidewire was then advanced into the tube and then the 3 French catheter was advanced into the tube over the guidewire. If the 0.018″ guidewire could not be advanced into the tube, a 0.025″ guidewire was used. After the 3 French catheter was advanced into the tube, the guidewire was removed and a selective salpingogram was done through the 3 French catheter. After the selective salpingogram was performed, the catheter was removed and a standard HSG was performed (Thurmond et al. 1987). Two of the patients had visualization of the tubes when selective salpingography was performed and so no further procedure was required. The other 3 patients had recanalization performed which was successful (Thurmond et al. 1987).

**Fig. 1:**
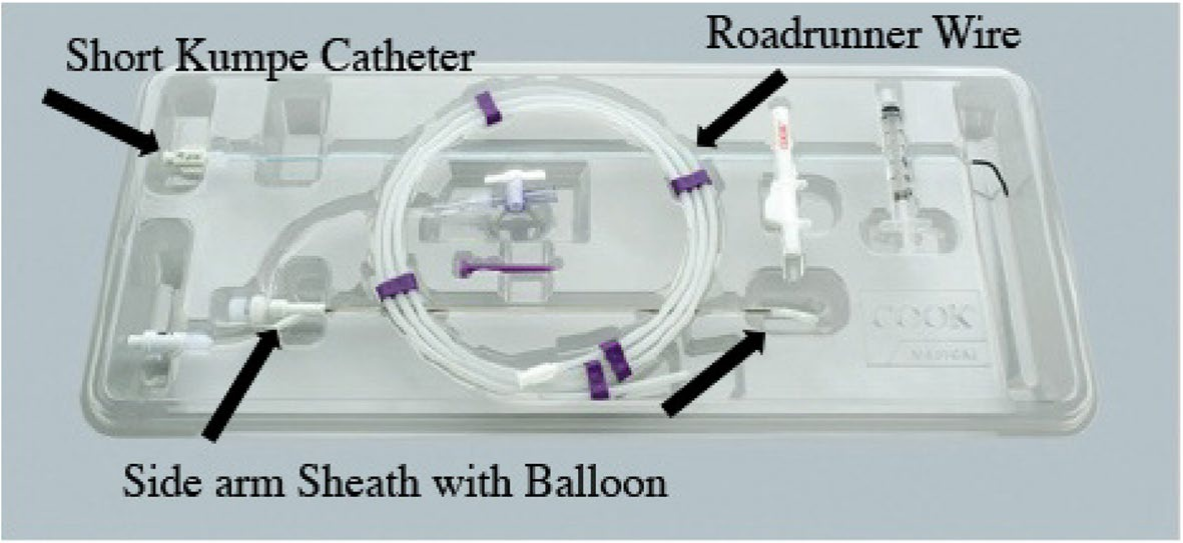
Fallopian tube recanalization kit previously made by Cook, Inc. (Cook Inc. Bloomington, IL). Although it is no longer available, it is a good illustration of the pieces of equipment which facilitate recanalization

**Fig. 2:**
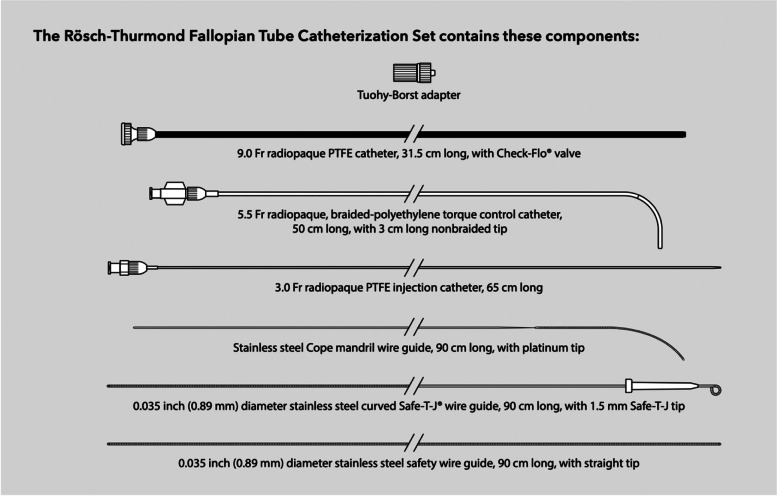
The components of the Rosch-Thurmond Fallopian Tube Catheterization Set (Cook Inc. Bloomington, IL). https://www.cookmedical.com/data/resources/RH-D56218-EN-F_M3_1594661982191.pdf

Multiple papers have been published over the last 35 years describing the procedure of proximal tubal recanalization using fluoroscopy. The technique has changed slightly, there has been development of various specialized equipment for performing the procedure, unfortunately one of the best recanalization sets (Radiographic Tubal Assessment Set by Cook Inc. Bloomington, IL) is no longer being offered by the manufacturer (Fig. 1). There is a Rosch-Thurmond fallopian tube catheterization set which is still available from Cook Inc. (Fig. 2). Although 0.018″ guidewires were commonly used early in the experience they have largely been supplanted with 0.035″ hydrophilic wires. The 0.018″ guidewire and microcatheter maybe used if one cannot cross the occlusion with a hydrophilic guidewire.

The tubal recanalization procedure may be performed as a stand-alone procedure following a HSG which was done at a previous setting. However, a combined procedure can be done beginning with an HSG and then if tubal occlusion is seen, a selective salpingography is done. If the tube is still occluded, then recanalization is attempted. Unilateral cornual occlusion can represent spasm or may represent the contrast taking the path of least resistance through the contralateral patent tube. Selective catheterization of the occluded cornua with a selective salpingogram done at the time of the HSG can help to verify the unilateral occlusion. Unilateral and bilateral proximal tubal occlusion should be evaluated with a selective salpingogram and if there is no filling of the tube, then recanalization can be performed.

## Tubal recanalization procedure

A tubal recanalization procedure begins with an HSG. The patient should be scheduled usually within 5–11 days of her menstrual cycle in the follicular phase of her cycle prior to ovulation (Thurmond et al. 2000). The patient should be pretreated with an antibiotic usually doxycycline 100 mg bid for 5 days starting 1–2 days prior to the procedure (Marlow et al. 2021). A urine pregnancy test is performed prior to the procedure. The patient is consented for the HSG and is told that if the tubes are patent, that will be the extent of the procedure, however, if one or both tubes are not visualized during the HSG then the plan would be for selective salpingography and possible attempt at recanalization if the selective salpingogram demonstrates proximal occlusion. Most patients are very happy to have the procedures combined, if necessary, since it avoids having to schedule another appointment for the attempt at recanalization. This allows for a one-step diagnostic and therapeutic procedure. It also exposes the patient to a single radiation dose rather than two separate radiographic procedures (Cobellis et al. 2012; Mallarini and Saba 2010).

Although the procedure is not particularly uncomfortable (Cobellis et al. 2012), many of the patients are extremely nervous, and anxious because of their struggles with infertility. In addition, some of the patients who have had previous HSGs may have had a great deal of pain, because of the pressure of the injection causing severe cramping particularly if they have bilateral tubal occlusions. Moderate sedation helps alleviate the anxiety and potential pain. Anecdotally, having moderate sedation potentially decreases the degree of tubal spasm allowing demonstration of tubal patency. Administration of a non-steroidal anti-inflammatory (NSAID) either oral or IV (Toradol) can be very helpful in decreasing cramping pain, and may help with decreasing the incidence of tubal spasm (Sowa et al. 1993). Perhaps most important is constant explanation of what the patient is going to be experiencing, and reassurance that things are going well.

**Fig. 3:**
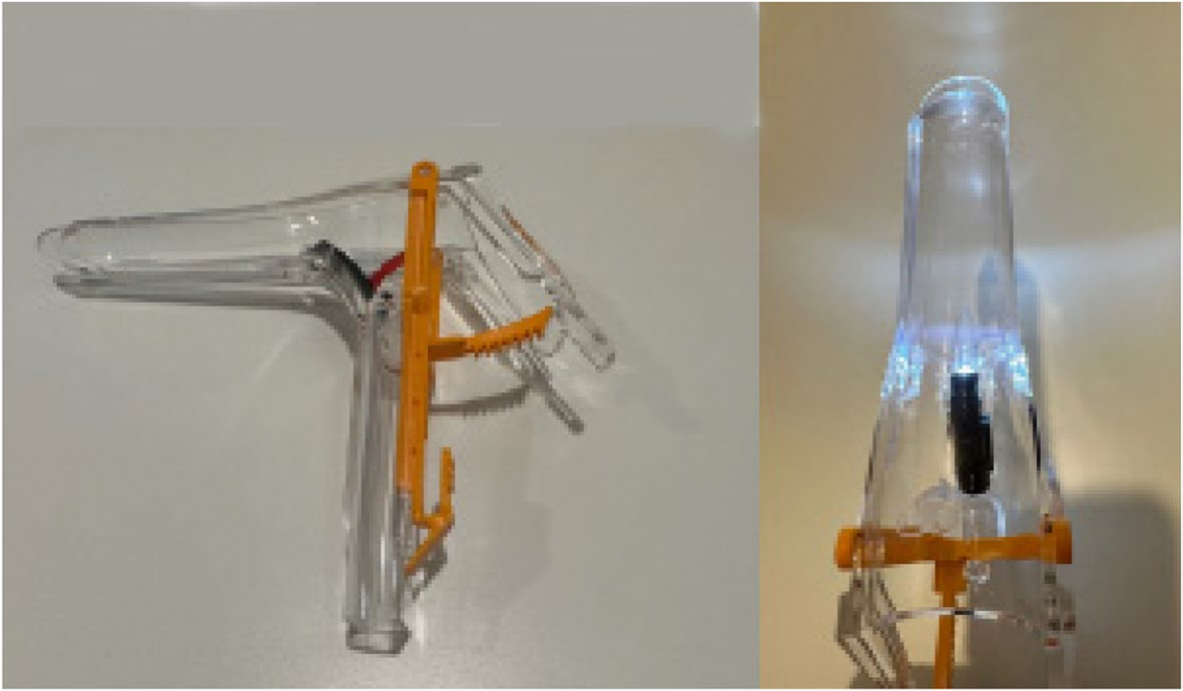
Plastic speculum with built in light source

**Fig. 4:**
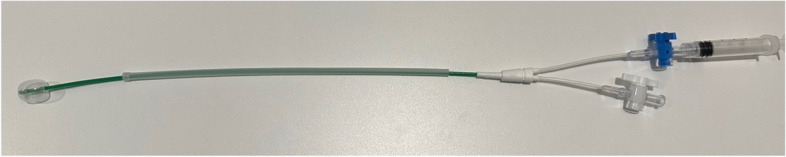
HSG catheter with balloon inflated. This catheter is 5.5 French. The plastic stiffener which helps stabilize it the catheter at the cervical os can be advanced into the cervix and the HSG catheter exchanged for a 5 French Kumpe catheter for selective salpingography and FTR. There are many types of HSG catheters on the market including some which are 9 French and have a lumen which will accommodate catheters up to 6.0 French

**Fig. 5:**
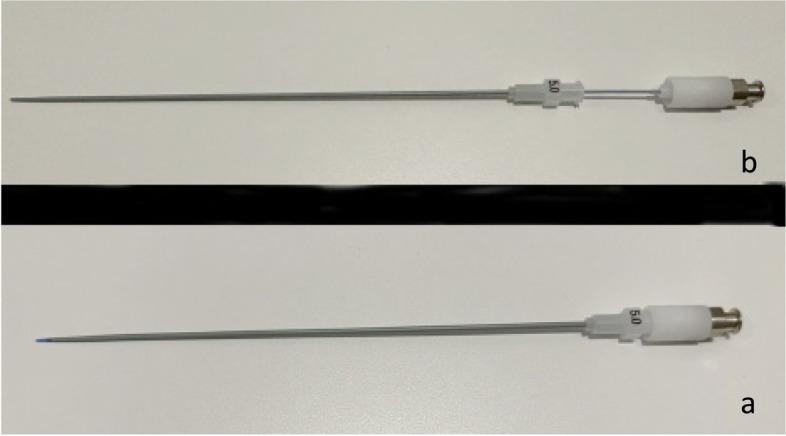
Micropuncture set (Cook Inc. Bloomington, IL). **a** shows the dilator and cannula placed together as one unit. **b** shows the dilator being pushed forward off the stiffened cannula

The patient is placed on a fluoroscopic table that is equipped with stirrups and placed in the lithotomy position. Following prepping and draping of the patient, a speculum (a plastic speculum with built in light is ideal) (Fig. 3) is placed, and the cervix prepped with Betadine. A standard HSG catheter with a balloon (Fig. 4) is placed in the uterine cavity and the balloon inflated. If there is difficulty in placing the balloon, a tenaculum can be used, but it is not usually necessary. At times if the uterus is markedly flexed a tenaculum can be used to straighten the uterus. If the catheter does not advance easily through the cervical canal, a 0.035″ hydrophilic guidewire (Glidewire, Terumo or Roadrunner, Cook Inc) can be placed through the HSG catheter, and advanced into the uterus, and then the HSG catheter can be easily advanced over the wire. This maneuver tends to make a tenaculum unnecessary. Occasionally there can be a very tight external cervical os which makes placement of the HSG catheter very difficult. A 4 or 5 French long (15 cm) stiffened micropuncture set (Cook Inc. Bloomington, IL) (Fig. 5) can be used to access the external os, and the 4 or 5 French dilator is gently pushed off the stiffened portion, the stiffened portion is removed, and a hydrophilic guidewire can be placed through the dilator into the uterine cavity. The dilator is then advanced into the uterine cavity, and a short 0.035″ Amplatz wire replaces the hydrophilic wire, then the cervical os can be dilated with fascial dilators, usually to 10–12 French. The HSG catheter can be placed over the wire, and the HSG performed.

**Fig. 6:**
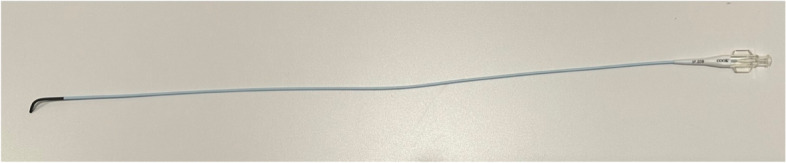
Kumpe catheter (Cook Inc. Bloomington, IL). The short (40 cm) length is ideal for this procedure

**Fig. 7:**
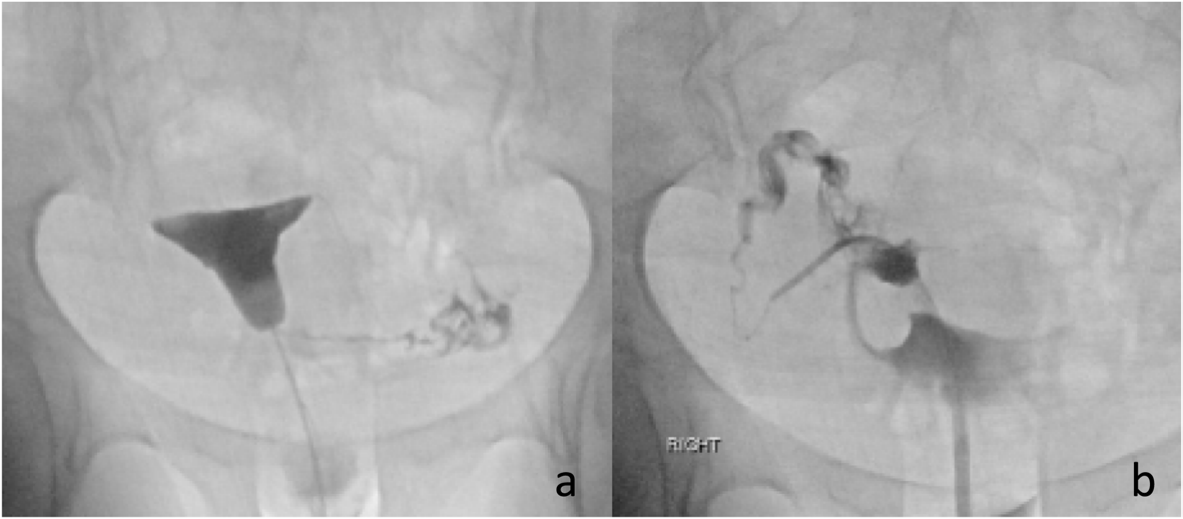
**a** shows filling of the left tube, but no filling on the right. In **b** the selective catheterization demonstrates a patent right tube

**Fig. 8:**
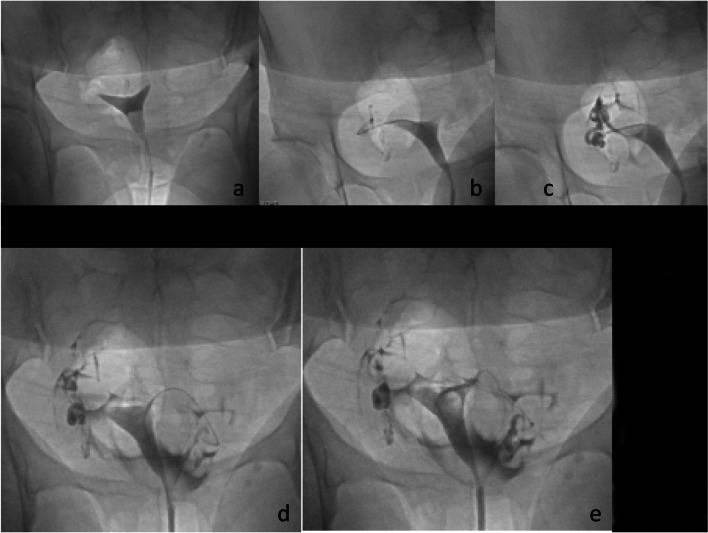
HSG in **a** shows occluded tubes bilaterally. **b** shows wire in proximal right tube. **c** shows patent right tube. **d** shows wire into the tube. **e** Shows patency of left tube

If the HSG demonstrates bilateral tubal patency, then the procedure is terminated after obtaining images of the uterus and fallopian tubes. If there is unilateral or bilateral proximal tubal occlusion, then a Kumpe catheter (Fig. 6) or other angled catheter, can be used to selectively catheterize the ostium of the tube. An injection of contrast is done, and visualization of the tube is attempted. In some cases, a selective injection may demonstrate filling of the tube. This is particularly common if there is unilateral occlusion and the contrast from the HSG is preferentially exiting from the patent tube (Fig. 7). Occasionally, the tube will be seen have a hydrosalpinx distally. If a hydrosalpinx is demonstrated, then care should be taken not to over distend the tube because of the risk of infection. If the tube continues to show proximal occlusion, then a 0.035″ hydrophilic guidewire can be placed and advanced a few centimeters into the tube. The catheter can then be advanced a millimeter or so into the ostium. After removing the guidewire another selective salpingogram is performed through the catheter (Fig. 8). Occasionally, the 0.035″ hydrophilic guidewire cannot be advanced through the tube, and attempts can then be made with a 0.018″ hydrophilic guidewire which is used in combination with a microcatheter, usually 2.8–3 French. After the recanalization of one or both tubes, another HSG can be performed to demonstrate patency of the tubes. To minimize the woman’s exposure to radiation, it is recommended to use digital, pulsed fluoroscopy, and static capture of the fluoroscopic images is obtained for documentation (Phillips et al. 2010). Radiation exposure to the ovaries is approximately 10 mGy estimated surface dose (ESD) for a normal HSG, but higher exposure to 47 mGy ESD for a selective catheterization (Thurmond et al. 2000; Phillips et al. 2010). The ovum is relatively radioresistant (Cobellis et al. 2012) so the risk from radiation is low.

Following the procedure, the patient should be told she may have some pelvic discomfort for a day or so, which responds well to anti-inflammatories. She should also be advised that some spotting may occur for 24–48 hours. She should also be counseled that sexual intercourse can be resumed the next day.

The results of transcervical fallopian tube recanalization (FTR) demonstrate a technical success rate of 70–100% (Marlow et al. 2021; Al-Omari et al. 2018; Thurmond et al. 2000; Wang et al. 2020) The intrauterine pregnancy rate has been reported as 20–60% with an average of 30% (Marlow et al. 2021; Thurmond et al. 2000) Patients with secondary infertility and shorter duration of infertility < 5 years may be more likely to achieve a pregnancy (Al-Omari et al. 2018). Age is a well-known risk factor for infertility, and this has been also shown to be a factor with FTR (Al-Omari et al. 2018). Patients younger than 35 have a higher pregnancy rate than those over the age of 35 (Al-Omari et al. 2018). The pregnancy success rate is higher in patients that only have proximal tubal occlusion, and normal distal tubes (Allahbadia and Merchant 2010; Sowa et al. 1993; Thurmond 1994). There was concern raised when this technique was in its infancy that guidewire cannulation alone was not an appropriate treatment for proximal tubal occlusion (Gleicher et al. 1994). It was argued there should be either tuboplasty or a coaxial recanalization which would consist of a 0.018 guidewire with placement of a 3 French microcatheter to allow for improvement in the luminal diameter. A study was done where recanalization was performed with only a 0.015 or 0.018″ guidewire (Gleicher et al. 1994). Tubal patency was demonstrated in 77% following wire placement, but the pregnancy rate was only 1 out of 24 patients and that was an ectopic pregnancy although not associated with the area of recanalization (Gleicher et al. 1994). It is possible that with the small caliber wire, the lumen was not enlarged enough, but with a standard 0.035–0.038″ wire used for recanalization this should not be an issue since a 3 French catheter is approximately equal to a 0.039″ guidewire.

Most pregnancies after catheter recanalization occur in the first 3–6 months (Thurmond 1994). If patients do not conceive within 6 months, it is recommended they undergo another HSG. Tubal patency at 6–12 months following FTR ranges from 40 to 72% (Thurmond 1994). If there is reocclusion then a repeat FTR can be performed. The repeat procedure has been successful in some patients at allowing them to achieve a pregnancy (Wang et al. 2020; Thurmond 1994).

A problem with many of the reports of tubal recanalization is they are primarily retrospective studies and may not control for other causes of infertility such as ovulatory or low ovarian reserve, male factors, and other issues which may impact fertility. They also tend to have small patient numbers. The reports are strongest in describing the technical success of the procedure but are likely to have missing data in the follow-up of patients who undergo tubal recanalization. The studies constitute a diverse group of patients and pregnancy rates have been variable, reflecting the diversity of the patient population (Thurmond et al. 2000).

One report by Al-Omari et al. (Al-Omari et al. 2018) was done as a prospective study. They had 61 patients who had infertility for a least 1 year, and prior HSG or laparoscopic examination demonstrating unilateral or bilateral tubal occlusion. Patients who had partners with male-related infertility factors, endometriosis, previous salpingectomy, ectopic pregnancy, pelvic inflammatory disease, or evidence of distal tubal blockage were excluded (Al-Omari et al. 2018). They had follow-up for up to 1 year and 41% of the women had conceived. The type and duration of infertility were significantly associated with pregnancy. Secondary infertility was associated with a 15-fold increase in the odds of conception, and infertility of < 5 years was associated with a 21-fold increase in conception (Al-Omari et al. 2018). They also found the pregnancy rates were higher in women younger than 35 years than in older women (Al-Omari et al. 2018).

There are some contraindications to performing selective salpingograms and FTR including infection, genital tuberculosis, and distal occlusions. Distal occlusions are more likely to be due to previous pelvic infection or endometriosis and are difficult to recanalize with poor pregnancy rates (Allahbadia and Merchant 2010; Kohi 2021). One type of distal occlusion, occlusion after tubal ligation reversal, may be attempted with FTR. There is one report of 24 patients with tubal obstruction after ligation reversal surgery and patency was established in 26 of the tubes 38 obstructed tubes (68%) (Thurmond et al. 1999). There were 7 uterine pregnancies and 2 tubal pregnancies reported in these patients (Thurmond et al. 1999). Salpingitis isthmica nodosa (SIN) can be attempted and has been successful in 77–82% of tubes but is technically more challenging (Thurmond et al. 2000). SIN maybe an underlying cause of inability to clear an obstruction (Sowa et al. 1993). There are very few patients with SIN that have had attempted recanalization and pregnancy rates are not known.

Complication rates of FTR are low with tubal perforation, infection and ectopic pregnancy reported in 1–9% (Marlow et al. 2021). Perforation occurs if the tubal obstruction is more fibrotic, and if the obstruction is more distal in the tube. It is recognized by contrast agent spilling into the peritoneal cavity (Thurmond et al. 2000). It is almost always asymptomatic, and occurs in approximately 2% (Kohi 2021; Thurmond 1994). Ectopic pregnancy can occur in approximately 3% of women (Thurmond 1994). All patients should be counseled that if they become pregnant that they need to have early evaluation to confirm that the pregnancy is intrauterine.

## Other techniques

Transcervical balloon tuboplasty has also been described. The first paper describing this technique was by Confino, et al. in 1986 (Confino et al. 1986). In this case report the procedure was performed using hysteroscopic visualization, as well as laparoscopic and fluoroscopic imaging. An 0.018″ guidewire was placed into the occluded tube, and then a 4 French angioplasty balloon catheter measuring 8 × 30 mm was advanced into the tube and dilated in the interstitial portion of the tube. A multicenter study of this technique was published, with 89 women with bilateral proximal tubal occlusions was published in 1990 (Confino et al. 1990). All of the patients had at least a year of infertility and previous HSG and laparoscopy demonstrating bilateral proximal tubal occlusion. In this report, the procedures were performed with only fluoroscopy, without hysteroscopy or laparoscopy guidance. A 2–3 mm balloon was advanced into the ostium and dilatation performed. Of the 89 patients, 18 (20%) achieved at least unilateral tubal patency during the HSG or the selective salpingography. Of these 6 underwent successful balloon recanalization of the remaining obstructed side. Twelve patients of the 18 patients (13% of the total number of patients) had bilateral tubal patency with either HSG or selective salpingography. Of the 77 patients that underwent transcervical balloon tuboplasty (TBT) 53 (69%) achieved bilateral patency and 18 (23%) achieved unilateral patency (Confino et al. 1990). Only in 6 patients (8%) was there failure to recanalize at least one fallopian tube (Confino et al. 1990). At the time of publication there had been 22 intrauterine pregnancies (34%) with 17 deliveries and 5 miscarriages. The rational for using balloon dilatation was that it would have the additional benefit of a functional sphincterotomy which might perform better than a procedure that only dislodges the intratubal plugs (Confino et al. 1990). The authors point out that determining if the balloon dilation is more effective would require a randomized study between dilatation and wire recanalization. Such a study does not seem to be reported, and balloon dilatation has the possibility of creating trauma in the small tubal lumen, thus, wire or coaxial recanalization would appear to be potentially less traumatic. Tuboplasty appears at this time to be primarily of historical interest.

Other methods of treating proximal occlusions have included endoscopic, ultrasound guided, hysteroscopic, open or laparoscopic surgical approaches or a combination (Allahbadia and Merchant 2010). As noted above, hysteroscopic guidance has been used to guide guidewire cannulation, and direct tuboplasty. It is commonly combined with laparoscopy to evaluate if there is spill from the tubes following recanalization. Hysteroscopic cannulation has a reported technical success rate of 40–80% for proximal occlusions with pregnancy rates reported from 13 to 54% (Allahbadia and Merchant 2010).

There has been the development of a endoscopic fiber catheter which can be placed transcervical and used to visualize the lumen of the fallopian tube, displace debris that may block the tube, breakdown intraluminal adhesions, or eliminate proximal endoluminal plaques (Allahbadia and Merchant 2010). This system consists of a hysteroscope, a flexible coaxial catheter and guidewire and a falloposcope with enhanced fiber optics (Allahbadia and Merchant 2010). Other devices require laparoscopy for evaluation (Tanaka et al. 2017). Another development is a linear everting catheter (LEC) which is used in combination with a microendoscope enables the visualization of the complete tubal mucosa from a transcervical approach (Allahbadia and Merchant 2010). This technique allows evaluation of the tubal lumen, tubal wall, and mucosa by direct visualization (Allahbadia and Merchant 2010). It also avoids the need for hysteroscopic guidance and so maybe performed with just moderate sedation. Recanalization success rates with these endoscopic recanalization techniques for proximal obstruction range from 40 to 94% with somewhat better results with the linear everting catheters. Technical success is what is most commonly reported, pregnancy rates have been reported at 22–27.6% (Allahbadia and Merchant 2010). The catheters available presently are prone to kinking and take some expertise and practice which limits their usefulness in routine clinical practice (Allahbadia and Merchant 2010).

Ultrasound can be used to guide transcervical recanalization using balloon tuboplasty, or cannulation with wire tuboplasty using laparoscopic control. Catheterization can also be guided by ultrasound with the use of an ultrasound contrast agent.

The surgical approach involved implantation of the most proximal fallopian tube into the uterus (Musich and Behrman 1983). This could be accomplished using cornual implantation, non-cornual implantation, or microsurgical anastomosis (Musich and Behrman 1983). The implantation techniques were described as a closed technique (tunneling the tube through a hole made by a reamer into the uterine cavity) or an open technique (incising the uterine wall to perform the implantation of the tube) (Musich and Behrman 1983). Given the anatomy of the UTJ it is understandable that these surgical procedures have been supplanted by transcervical recanalization or in vitro fertilization.

## Conclusions

There has been significant progress in the treatment of infertility caused by proximal tubal occlusion over the last 35 years. Fluoroscopic guidance for selective salpingography and recanalization is an effective and minimally invasive way to treat proximal occlusions. It avoids the need for surgical laparoscopic guidance, and the cervical dilatation needed for hysteroscopic guidance, and does not require the administration of anesthesia. By combining the diagnostic HSG with the ability to perform selective salpingography and recanalization the patient is spared two procedures. Selective salpingography with tubal recanalization is not just a therapeutic procedure, it can be diagnostic as well. Even when FTR is unsuccessful, it can provide helpful diagnostic information which can influence the treatment plan for treating the infertility. Transcervical tubal recanalization procedures, which are low-risk, and cost-effective, can provide an excellent alternative to invasive and expensive surgical procedures and assisted reproductive technologies. The American Society for Reproductive Medicine has recommended that patients who have proximal tubal obstruction undergo selective salpingography and tubal recanalization before considering more invasive and costly treatments (Thurmond et al. 2000; Practice Committee of the American Society for reproductive medicine. Electronic address Aao 2021).

## Data Availability

PubMed search for articles on fallopian tube recanalization. No specific data base used.

## Abbreviations

UTJ: uterotubal junction
HSG: Hysterosalpingogram
ESD: Estimated Surface Dose
IFTO: Interstitial fallopian tube obstruction
SIN: Salpingitis isthmica nodosa
FTR: fallopian tube recanalization
NSAID: Non-steroidal anti-inflammatory
TBT: transcervical balloon tuboplasty
LEC: linear everting catheter
